# Practical aspects in the management of hypokalemic periodic paralysis

**DOI:** 10.1186/1479-5876-6-18

**Published:** 2008-04-21

**Authors:** Jacob O Levitt

**Affiliations:** 1 President and Medical Director, Periodic Paralysis Association, 155 West 68th Street, #1732, New York, NY 10023, USA; 2 Assistant Clinical Professor, Department of Dermatology, The Mount Sinai Medical Center, New York, NY, USA

## Abstract

Management considerations in hypokalemic periodic paralysis include accurate diagnosis, potassium dosage for acute attacks, choice of diuretic for prophylaxis, identification of triggers, creating a safe physical environment, peri-operative measures, and issues in pregnancy. A positive genetic test in the context of symptoms is the gold standard for diagnosis. Potassium chloride is the favored potassium salt given at 0.5–1.0 mEq/kg for acute attacks. The oral route is favored, but if necessary, a mannitol solvent can be used for intravenous administration. Avoidance of or potassium prophylaxis for common triggers, such as rest after exercise, high carbohydrate meals, and sodium, can prevent attacks. Chronically, acetazolamide, dichlorphenamide, or potassium-sparing diuretics decrease attack frequency and severity but are of little value acutely. Potassium, water, and a telephone should always be at a patient's bedside, regardless of the presence of weakness. Perioperatively, the patient's clinical status should be checked frequently. Firm data on the management of periodic paralysis during pregnancy is lacking. Patient support can be found at .

## Introduction

Hypokalemic periodic paralysis is a disorder of muscle whereby voltage-gated ion channels (typically calcium or sodium, and less frequently potassium) are mutated, resulting in abnormalities of sarcolemmal excitation. The disease typically first manifests in adolescence as bouts of mild to severe muscle weakness lasting hours and sometimes days associated with hypokalemia triggered most commonly by rest after exercise or high carbohydrate meals. Weakness typically recovers when serum potassium normalizes. In some cases, a fixed weakness can develop, especially in the larger, proximal muscle groups later in life. Lehmann-Horn et al provide a detailed overview of periodic paralysis pathophysiology elsewhere [1].

Being Medical Director of the Periodic Paralysis Association and myself a carrier of the R672S mutation in Nav1.4, encoded by SCN4A (my case was reported by Venance et al [2]), has allowed me to compare the experiences of scores of patients who suffer from muscle ion channelopathies. Through my own experience with primary hypokalemic periodic paralysis, as well as through numerous anecdotes of other sufferers, I have collected some useful insights in dealing with the disorder on a day to day basis. To verify each of these management strategies in a scientifically rigorous way is important but impractical due to the rarity of the periodic paralyses. As such, a good portion of this manuscript represents my personal observations in the management of hypokalemic periodic paralysis.

## Discussion

### Potassium dosing and indications

While one could make a physiologic argument for one or another type of potassium salt, in all likelihood, the counter ion is unimportant. It is felt that potassium chloride is best absorbed [3], making this the favored salt in an acute attack. Also, metabolic alkalosis frequently accompanies hypokalemia and Cl^- ^best corrects the alkalosis [4]. That being said, each vehicle – be it oral solution, powder dissolved in water, or sustained release tablet – has its place in management. Oral solution, as measured in mg/mL, can be as effective as powder in water; however, it is cumbersome to convert milligram (mg) dose to mEq dose, and confusion can arise because of this. Indeed, depending on the counter ion, one mg of potassium salt can translate into different mEq amounts. To be safe and consistent, *always dose in milliequivalents *(mEq). Table 1 is useful for conversion to mEq. Various brands contain combinations of salts, and in these cases, it is critical to read the package insert if dosing on a mg basis rather than a mEq basis.

**Table 1:** Potassium salts and their milligram to milliequivalent (or millimole) relationships [2]

Spoken name	Chemical Formula	mg	mEq K+ (or mM K+)
Elemental potassium ion	K+	39.1	1
Potassium chloride (powder or tablets)	KCl	1500	20
Potassium bicarbonate (powder, tablets, or self-dissolving tablets)	KHCO_3_	650	6.5
Potassium citrate monohydrate	K_3_C_6_H_5_O_7*_H_2_O	540	15 (i.e 5 mEq of K_3_C_6_H_5_O_7_)
Potassium chloride 10% oral solution, 15 mL	KCl 10% oral solution	1500	20
Potassium chloride 20% oral solution, 15 mL	KCl 20% oral solution	3000	40
Potassium gluconate elixer, 15 mL	KC_6_H_11_O_7_	4680	20
Potassium gluconate tablets	KC_6_H_11_O_7_	500	2

With respect to liquid/aqueous versus sustained release tablet, liquid and aqueous forms of potassium are useful prior to performing known triggers, such as eating a high carbohydrate load and vigorous activity or at the start of an episode of weakness. (Once the vigorous activity is over and rest begins, an attack will often occur without appropriate prophylaxis). In cases where the patient has frequent early morning attacks, a trial of sustained release potassium tablets taken at time of sleep may be warranted. Some may also find it necessary to take sustained release tablets once or twice daily as a standing regimen in combination with immediate release potassium as needed. The downside to chronic potassium administration, especially with the use of sustained release tablets, is gastric irritation from locally high potassium salt concentrations. This problem is often well-controlled with a proton pump inhibitor.

The vehicle in which to mix the potassium salt warrants consideration. Water is the favored option. Sports drinks, such as Gatorade^®^, may at first blush seem intuitively beneficial as they contain "electrolytes" and "potassium"; however, they contain high sugar loads and often high sodium content, both of which can trigger attacks in hypokalemic periodic paralysis patients. Potassium should be taken with ample volumes of water, for example, at least 120 mL (about 4 oz) of water per 20 mEq [3]. I have found that dissolving the potassium in the least amount of liquid possible, ingesting in one shot, and following with an 8–12 oz water chaser is more tolerable than drinking a full 8–12 oz of aqueous solution.

Potassium can be given orally for attack prophylaxis. Assuming normal renal function, most patients are grossly under-dosed. One can find the appropriate dose by trial and error, or approach it more scientifically. Either way is acceptable. The scientific approach involves measuring serum potassium 20 to 40 minutes after fixed oral potassium loads on separate days. This should provide some confidence to both patient and physician as to what is safe and what may be dangerous. For example, it is not uncommon for a 60 kg individual to require 60 mEq of potassium for attack prophylaxis prior to vigorous exercise. Using an aqueous form (rather than sustained-release tablets) allows for an appropriate peak in serum potassium concentration and should be taken no more than one hour prior to exercise. Trial and error will allow the individual patient to find his appropriate therapeutic window with respect to dose and timing of dose. It is prudent to try different doses prior to exercise, starting low and titrating up prior to each exercise session until the minimum dose that prevents paralysis is found. Repeated periods of exercise spaced as little as three hours apart may require additional prophylaxis. For example, one may need an additional 60% of the first dose taken that day.

The danger of overdosing on oral potassium is low [3], provided that the dose is within reason. A good rule of thumb is not to exceed 100 mEq in a day in the absence of an attack. Renal excretion of potassium is 90% and fecal excretion is 10%. Renal excretion becomes more robust with chronic potassium dosing [3]. Before cardiac side effects would occur, osmotic diarrhea and paresthesia of the lips, hands, and feet typically intervene. This would be an indication to limit the potassium intake. Patients should be educated as to the safe and appropriate use of their potassium; however, they should never be restricted as to the amount of potassium supplement available to them. One month's prescription should be able to accommodate the assumption that the patient will use their maximum daily dosage each day of that month.

Oral potassium administration, assuming normal renal function, is the mainstay for treating attacks of hypokalemic paralysis. Here too, most patients are grossly under-dosed with respect to potassium supplementation during an attack. Large bolus doses are favored over smaller, spaced out, incremental doses. As a general rule, 40 to 60 mEq K^+ ^raises plasma potassium concentration by 1.0 to 1.5 mEq/L, and 135 to 160 mEq K^+ ^raises plasma potassium by 2.5 to 3.5 mEq/L [4]. For a 60 to 120 kg man, 60 mEq is a reasonable initial dose (i.e. 0.5 – 1 mEq/kg). Arriving at the optimal dose for any one individual will initially be a process of trial and error. For example, some patients find that 0.5 mEq K^+^/kg reproducibly resolves attacks, and others find they need 1 mEq K^+^/kg. Of course, the dose will vary from attack to attack depending on the severity of its associated hypokalemia. Unfortunately, patients usually do not have the benefit of a serum potassium reading when faced with the decision of what dose to take.

Quicker results are obtained using aqueous potassium, and this is the favored form for acute attacks. Partial, if not complete, response should be seen within 30 minutes. If no improvement is seen after 30 minutes, an additional 30% dose should be given – that is, an additional 0.3 mEq/kg. This process should proceed up to roughly 100 mEq. If an attack has not resolved after 100 mEq of potassium, it is prudent to monitor the serum potassium prior to further supplementation in an acute care setting. Typically, the total dose of potassium should not exceed 200 mEq in a 24 hr period in the treatment of an acute attack.

Intravenous potassium should be avoided whenever possible; however, it is indicated for arrhythmia due to hypokalemia or airway compromise due to ictal dysphagia or accessory respiratory muscle paralysis. Mannitol (which is inert) should be used as the solvent (rather than saline or dextrose, which are both potential triggers of attacks) [5]. One must be cautious to give only 10 mEq at a time, separated by anywhere from 20–60 minutes, as there is a distinct danger of overshooting the mark, resulting in hyperkalemia. That is, the first aim is to get the patient out of danger rather than attempting rapid, complete correction [4]. Patience, provided that the patient is stable, is important. While 10 mEq per hour is the favored rate [4], urgent situations such as severe arrhythmia or respiratory compromise may warrant temporary, higher rates of administration [3]. The hypokalemia in hypokalemic periodic paralysis is due to potassium shifts from blood to muscle. When the attack resolves, potassium returns to the blood. So, the idea is to administer just enough potassium to induce physiologic resolution of the attack.

Patients will often complain of a burning sensation due to potassium irritation of the recipient vein. There are anecdotal reports of intravenous lidocaine being used to mitigate the burning; however, the safety of this strategy has not been established. If larger doses than 10 mEq at one time are needed, a larger vein should be used, sometimes requiring a femoral venous line [4]. Furthermore, no more than 80 mEq per liter, and preferably no more than 40 mEq per liter, should be used, even in a central vein [4,5].

### Maintenance therapy

While potassium can be taken chronically as maintenance, the favored approach is to add one of a variety of diuretics, some of whose mechanisms in periodic paralysis are poorly understood. *Diuretics are not helpful for acute attacks*. Their role is to decrease the frequency and severity of attacks over time. The first line approach is acetazolamide, dosed anywhere from 125 mg to 1000 mg per day in divided doses, either in immediate or sustained release preparations [6]. Finding the correct dose requires some trial and error, with 250 mg twice daily being a reasonable starting point. Some patients have worsening of the paralytic attacks with acetazolamide. Others complain of paresthesias and cognitive impairment (described by patients as mental dullness, fogginess, inability to concentrate, or confusion) from acetazolamide. Acetazolamide-induced nephrolithiasis (calcium phosphate stones) is not uncommon [7,8] and can often be controlled with dose reduction and appropriate hydration under consultation with a urologist.

Because carbonic anhydrase inhibitors are potassium-wasting, occasional potassium supplementation, even in the absence of an attack, may be warranted. If symptoms worsen with acetazolamide, they likely will not improve with dichlorphenamide. However, if the patient has no response with acetazolamide or if symptoms were once controlled with acetazolamide but progress to become refractory to acetazolamide, then dichlorphenamide is a good option. Dichlorphenamide is also a reasonable first-line therapy; however, it is more potent than acetazolamide and issues with mental confusion and cognitive impairment tend to be more frequent than with acetazolamide [6]. Dosing is based on the premise that dichlorphenamide is five times as potent as acetazolamide, such that a 250 mg dose of acetazolamide corresponds to a 50 mg dose of dichlorphenamide [6].

If carbonic anhydrase inhibitors are not an option, aldosterone antagonists can be useful. Specifically, spironolactone is commonly used with good success since it is a potassium-sparing diuretic. Side effects of gynecomastia make this a less favored treatment for males. Epleronone is a novel aldosterone antagonist that reportedly has fewer issues with gynecomastia while retaining its potassium-sparing properties. It is also FDA Pregnancy Category B. As such, this should be tried before spironolactone. It is safe to take potassium supplementation and be on an aldosterone antagonist in the case of hypokalemic periodic paralysis. However, the patient and physician must work together to find the minimum amount of potassium required while on the aldosterone antagonist to prevent or abort an attack without causing hyperkalemia. Blood pressure should be monitored when making dose adjustments.

When one agent or another agent does not adequately control the frequency and/or severity of attacks, combination therapy of a carbonic anhydrase inhibitor (provided it does not cause disease exacerbation) and an aldosterone antagonist may be tried. Other less frequently used potassium-sparing diuretics that can also be tried are triamterene and amiloride. Electrolytes should be monitored initially every two weeks, and monthly or quarterly thereafter.

### Behavioral and environmental management, trigger identification, and diet

A safe bedside environment must be established for the periodic paralysis patient. Frequently, a patient will awake during the early morning with enough movement to self-medicate and call for help before progressing to complete paralysis. If the patient is fortunate enough to still have movement, he must: a) call for help and alert someone that he is paralyzed, and b) immediately take his potassium prior to further activity. This requires the following at the bedside: a telephone, a plastic bottle of water that is easily opened, potassium, a plastic or paper cup in which to mix the medicine, and a spoon and/or straw to mix the medicine. Glass should not be kept at the bedside because patients have lack of coordination and strength due to paralysis and risk dropping and breaking the glass. There should be no slippery rugs at the foot of the bed, as patients sometimes need to maneuver in a partially paralyzed state and cannot balance themselves in the event of lost traction. Many patients have suffered bone fractures from falling while paralyzed. Finally, the bed should not be near an air conditioning vent in the summer or near a window during winter, as cold during the night can induce an attack, either as a trigger unto itself or by inducing shivering, which constitutes exercise that results in an attack.

The time from premonitory symptoms to full blown attack is often very short, on the order of minutes. For that reason, a sufficient dose of potassium to prevent and relieve an attack should be kept in various places at all times, including: at the bedside, in a pocket of coat, in the car, in the pocketbook, and in the suitcase. Potassium should be kept on the person at all times.

One helpful exercise, especially for those individuals experiencing a high frequency of attacks, is to make a diary of attacks. They should document whenever they get an attack, paying attention to what they ate within the 24 to 36 hours preceding an attack, what activities they did within the preceding 24–36 hours prior to the attack, and what medications they took or forgot to take. As some triggers are completely individual, they are only identified by careful attention to the details surrounding the attacks. Once triggers are identified, the patient should avoid them. Sometimes one can relate a series of attacks over consecutive days to one large triggering event.

Identifying and avoiding triggers of attacks is imperative. Numerous triggers have been reported to induce attacks of paralysis. The most consistent are rest after exercise and high carbohydrate meals. A high carbohydrate meal causes a spike of endogenous insulin release, resulting in a potassium shift from outside to inside myocytes. The potassium shift triggers inappropriate activity of mutated voltage-gated ion channels, resulting in membrane depolarization, and manifesting as paralysis.

Sodium intake triggers some, but not all, patients. It is important for salt-sensitive patients to discover the amount of salt that induces attacks for them. This amount varies from person to person, and indeed, from day to day. Patients must be aware of the amount of salt in the foods they eat, preferably prior to eating them. For those patients that like to salt their food, potassium chloride (available as NoSalt^®^), rather than sodium chloride, can be used.

The remaining list of triggers vary for individual patients, but patients and doctors should be aware of what types of things to look out for: cold, upper respiratory infections (both viral and bacterial), fever, lack of sleep/fatigue, Chinese food – with or without monosodium glutamate (presumably a trigger because of high starch content), alcohol, dehydration, startle, any medications – some with a physiological explanation (insulin, beta-agonists, corticosteroids [9]) and many without (determined by trial and error), menstrual cycle, change in humidity or barometric pressure, and change in daily activity patterns (i.e., going from regular exercise one week to being sedentary the next week and vice versa).

Patients are sometimes unclear as to what constitutes a "high carbohydrate food," and these must be outlined explicitly to the patient. One woman got paralyzed from "coffee" but did not equate the three teaspoons of sugar she added with the paralysis trigger. Pastas, candy bars, sports and energy drinks, cola drinks, orange juice, apple juice, pizza, bread, rice, potatoes, and beer (aside from alcohol content) are all sources of high carbohydrates. Some people have had success with buckwheat and other sources of more complex carbohydrates.

A common misconception is that one can increase banana intake to avoid paralysis. A medium-sized (118 g, 7 1/2 inch-long) banana contains 467 mg of potassium, or 12 mEq of potassium (not all of which would be bioavailable), and 28 g carbohydrates [10]. One would need to eat six bananas to get 72 mEq of potassium, which is a significant (168 g) carbohydrate load and constitutes roughly 1.5 lbs. worth of bananas!

### Intercurrent illness, surgery, and pregnancy

Diarrhea is potassium-wasting, and can precipitate attacks of paralysis. Some patients are challenged when the diarrhea is a result of too much oral potassium intake over a short period of time. In this case, further supplementation should probably not be done without monitoring of serum potassium. Influenza can cause some people to be particularly susceptible to attacks; that is, for a given trigger, the threshold of attack is lower during an upper respiratory infection than during health. Insulin-dependent diabetes mellitus is a particular challenge, in that insulin drives potassium into cells, precipitating hypokalemia and an attack of paralysis. Indeed, insulin can be used as a diagnostic challenge test; however, the glucose-insulin challenge test is discouraged due to risk of both profound paralysis and life-threatening hypoglycemia.

Periodic paralysis patients have special needs with respect to both minor and major surgery. The gene, CACN1AS, in which mutations cause primary hypokalemic periodic paralysis, is allelic to that which causes malignant hyperthermia susceptibility [11]. Malignant hyperthermia is characterized by generalized muscle rigidity, rhabdomyolysis with elevated creatine kinase, hypercarbia, acidosis, hyperkalemia, and hyperthermia after exposure to inhalation narcotics or depolarizing muscle relaxants [12]. Genetic linkage of malignant hyperthermia and hypokalemic periodic paralysis has never been definitively demonstrated [13]; however, surgeons and anesthesiologists should be aware of this possibility and be prepared to deal with it. More often, patients will simply become paralyzed during or after surgeries, as the surgical theater is cold, the surgery itself is a stressor, and the use of triggering agents (i.e., dextrose and saline) is often unavoidable. Difficulty with extubation can theoretically be due to an attack of paralysis. As such, it is prudent to monitor potassium somewhat more carefully in the peri-surgical period as well as to check on the patient frequently. Another possibility, albeit unlikely, is inadequate anesthesia during surgery, whereby paralysis from an attack rather than from pharmacologic neuromuscular blockade is mistaken for successful anesthesia. With respect to minor surgeries or procedures in the primary care, dermatology, ophthalmology, or dental settings, intralesional lidocaine with epinephrine or beta-agonist eye drops can cause sudden and severe attacks of paralysis in some individuals. If this is a known complication, measures to avoid these agents should be taken where possible. For example, one can use lidocaine without epinephrine. One opthalmologist successfully employed lacrimal duct plugs to avoid absorption of dilating eye drops.

Management of periodic paralysis during pregnancy and during parturition is challenging. Potassium management should be no different from prior to pregnancy. Carbonic anhydrase inhibitors like acetazolamide and dichlorphenamide are FDA Pregnancy Category C. Their use must be weighed against the risk of not taking them. Many women choose to avoid carbonic anhydrase inhibitors for the duration of their pregnancy. For some, this is tolerable, for others, it is exceedingly challenging. Of note, eplerenone is FDA Pregnancy Category B and is the favored option for those patients who cannot manage on potassium supplementation alone.

### Diagnosis

Accurately diagnosing channelopathies is difficult to the uninitiated (and to the initiated!). Aside from the classical periodic episodes of weakness in response to triggers, patients often report additional symptoms either before, during, or after attacks that may not have good physiologic explanations. These include prodromal paresthesias, sweating [12], post-ictal myalgia [14], post-ictal extreme fatigue, thirst, shortness of breath (either due to anxiety or to the episode itself), palpitations, clumsiness, irritability, and mental dullness. The physical exam is often normal. The neurological strength exam itself can trigger an episode, and some patients have reported leaving a doctor's office very weak from the sudden, unexpected muscle work of the exam.

The list of differential diagnoses (Table 2) stems from three basic considerations: a) exercise-induced symptoms of either stiffness or weakness, b) recurrent bouts of hypokalemia, and c) episodic muscle weakness. The presence of the following disorders does not exclude, can coexist with, and should NOT preclude treatment of periodic paralysis, when present, and vice versa: chronic fatigue syndrome, fibromyalgia, depression, personality disorder, psychosomatization, and malingering (often NOT the case, but a diagnosis that is all too frequently and inappropriately given). In the absence of readily available genetic testing or dangerous provocative testing, the diagnosis often can be identified with a judicious combination of considerations (Table 3).

**Table 2:** Differential Diagnosis of Channelopathies

Thyrotoxic hypokalemic periodic paralysis (TPP)
Primary (or familial) hypokalemic periodic paralysis (hypoPP)
Hyperkalemic periodic paralysis (hyperPP)
Paramyotonia congenita (PMC)
Potassium-aggravated myotonia (PAM) [22, 23] (includes myotonia fluctuans [24], acetazolamide-responsive myotonia [25], and myotonia permanens [26])
Myotonia congenita, both recessive and dominant (MC)
Andersen-Tawil syndrome (ATS) (formerly Andersen syndrome)
Hyperaldosteronism and physiologically similar states
Renal tubular acidosis Type IV (RTA)
Diuretic abuse
Myasthenia gravis

**Table 3:** Some Historical, Physical Exam, and Laboratory Diagnostic Considerations for Ion Channelopathies [9,16]

Question	If positive, suggests:
Family history	hyperPP, hypoPP, ATS, PMC, MC, PAM
Carbohydrates induce attacks	TPP, hypoPP, +/- PMC, ATS
Carbohydrates ameliorate attacks	hyperPP, ATS, PMC, PAM
Stiffness after exercise	PMC, MC
Tongue stiffens when eating ice cream	PMC
Less stiffness decreases with repeated exercise of a given muscle (warm-up phenomenon)	MC
Myotonia increases with continued exercise	PMC
Serum potassium elevated during attack	PAM, hyperPP, ATS, PMC
Serum potassium normal during attack	all diagnoses are possible
Serum potassium low during attack	hypoPP, TPP, ATS, PMC, diuretic abuse, hyperaldosterone states, RTA
Difficult to open eyes in the cold	PMC
Attacks of muscle stiffness	MC, ATS, PMC, PAM
Attacks of muscle weakness	MC, TPP, hyperPP, hypoPP, ATS, PMC
Duration of attacks are hours	hypoPP, TPP, ATS, PMC
Duration of attacks are minutes to hours	hyperPP, PAM, MC, ATS
EMG with myotonia	hyperPP, PAM, MC
EMG silent during attack of weakness	hypoPP, TPP, ATS, PMC, MC
Palpitations	ATS, hypoPP, hyperPP, TPP, PMC
EKG – tachycardia	TPP
EKG – long QTc and/or ventricular arrhythmia	ATS
EKG – u waves	ATS, hypoPP, TPP
Hyporeflexia during attack of weakness	hypoPP, TPP, ATS, hyperPP
Percussion myotonia	MC, PMC, PAM
Physical exam with some of: fifth digit clinodactyly, ocular hypertelorism, low-set ears, webbed fingers/toes, broad nasal root, small mandible, short stature, brachydactyly, microcephaly, short palpebral fissures, thin upper lip, small hands/feet, residual primary dentition, delayed bone age [16]	ATS
McManis nerve conduction protocol (i.e., changes in compound muscle action potential after exercise)	ATS, hyperPP, hypoPP, TPP
Muscle biopsy with tubular aggregates	ATS, hyperPP, hypoPP, TPP, PMC, PAM, MC

hyperPP = hyperkalemic periodic paralysis; hypoPP = hypokalemic periodic paralysis; ATS = Andersen-Tawil syndrome; PMC = paramyotonia congenita; MC = myotonia congenita; PAM = potassium-aggravated myotonia; TPP = thyrotoxic periodic paralysis; RTA = renal tubular acidosis

Provocative testing can be dangerous and is not a favored first-line method of diagnosis. Potassium challenge tests risk hyperkalemic arrhythmia, even in an acute care setting. Insulin challenge tests can be equally dangerous due to risk of hypoglycemia. Simple exercise challenge, which is relatively safe, is partly helpful when the ictal serum potassium is high or low. The distinction of hyperKPP versus hypoKPP can often be made, but the presence of ATS would still be unresolved. In fact, hyperkalemic and hypokalemic challenges should never be done if there is a suspicion of ATS. Also, an equivocal challenge test does not rule out one or another disorder.

Both hyperkalemic and hypokalemic periodic paralysis can present with normokalemia during attacks. Potassium challenge in hyperkalemic periodic paralysis would induce an attack. Potassium administration in hypokalemic periodic paralysis makes the attack resolve. Furthermore, it is helpful in some cases to compare the ictal potassium with the patient's baseline. Sometimes both ictal and baseline are within normal limits but ictal is either lower or higher than baseline, rendering a clue as to the nature of the periodic paralysis.

Various laboratory examinations (outside of ictal and interictal serum potassium measures) play an important role in both diagnosis and management of hypokalemic periodic paralysis. Specifically, EKG, TSH, free T3, and free T4 are the minimum indicated labs, with renal and adrenal function also recommended. Thyroid function evaluation will identify thyrotoxic periodic paralysis, which should resolve with the achievement of euthyroid status. Renal and adrenal axis screening is helpful to rule out hyperaldosterone states, renal tubular acidosis type IV, and diuretic abuse. Perhaps most important is the EKG, as patients with ATS typically have a long QTc interval (that is frequently overlooked or dismissed) and/or a variety of ventricular arrhythmias, including polymorphic ventricular tachycardia, bidirectional ventricular tachycardia, and ventricular bigeminy. One patient with Wolff-Parkinson White symdrome and primary hypokalemic periodic paralysis has been reported [15], but this is likely a coincidental association. Taking an EKG both during asymptomatic periods with normal serum potassium and during an attack (when potassium levels are low) is considered standard of care. Oftentimes, arrhythmias in this disorder are uncovered during the attack; whether or not they require treatment is a difficult judgement call.

The role of genetic testing is becoming increasingly important for cases with a high pretest probability of being a neuromuscular ion channelopathy. Not only can genetic testing reliably distinguish hyperPP from hypoPP and ordinary periodic paralysis from ATS, it also has some bearing on prognosis. For example, patients with hypoPP related to sodium channel mutations have not demonstrated permanent muscle weakness (PMW) whereas those patients with calcium-channel related mutations are at greater risk for PMW [16]. To date, other clinically useful genotype-phenotype correlations are lacking [16,17]. Genetic testing has very high specificity but poor sensitivity. A negative genetic test does not rule out the presence of periodic paralysis, as there are believed to be other, as yet unidentified, mutations and channels that are responsible for the clinical manifestations of these disorders. A positive test, on the other hand, is confirmatory and extremely informative. Genetic testing is available on a research basis in various centers around the world.

With respect to genetic counseling, these disorders are autosomal dominant with decreased penetrance in some females. The predeliction for Asian males relates only to the acquired, thyrotoxic form of hypokalemic periodic paralysis. Nearly all patients with periodic paralysis live long, productive, and fulfilling lives.

### Patient support

There are several patient support organizations for periodic paralysis patients. The most relevant are the Periodic Paralysis Association (PPA), the Periodic Paralysis News Desk, and the Muscular Dystrophy Association (their web addresses can be found in the References section below) [18-20]. The PPA is a non-profit charitable corporation founded in recognition of the need for an organization that fosters awareness of the periodic paralyses, promotes science-based information regarding this class of disorder, and champions the interests of the Periodic Paralysis Community. The Periodic Paralysis News Desk provides information on the periodic paralyses for patients, their families and caregivers, and physicians. Importantly, this website is available in Spanish. Both organizations contain listservs for patients to share their experiences with all aspects of this and other neuromuscular ion channelopathies. The PPA has created an on-line patient registry [21], whose aim is to aid in periodic paralysis-related research.

## Conclusion

A myriad of questions surrounding management of this condition remains unstudied. Of particular significance are: 1) What percent of patients will go on to permanent muscle weakness, and are there markers for this risk? 2) Is permanent muscle weakness preventable? 3) Does strict control of attacks influence development of permanent muscle weakness? 4) Are there genotype-phenotype correlations with respect to medication success, triggers, comorbidities, and disease course? 5) Can patients develop insensitivity to one or another carbonic anhydrase inhibitor over time? 6) Are there risks to chronic potassium administration? 7) What is the optimal dosing strategy for potassium during an attack? 8) Does a particular form of potassium work better than another for this disease? 9) What is optimal management during pregnancy? Studies are needed to validate the diagnostic and management strategies that are currently in practice. It is hoped that with the combined efforts of various researchers and patient support organizations, a large enough cohort of genetically characterized patients and families can be collected to answer these questions.

## Abbreviations

hyperPP: hyperkalemic periodic paralysis; hypoPP: hypokalemic periodic paralysis; ATS: Andersen-Tawil syndrome; PMC: paramyotonia congenita; MC: myotonia congenita; PAM: potassium-aggravated myotonia; TPP: thyrotoxic periodic paralysis; RTA: renal tubular acidosis.

## Competing interests

The author declares that he has no competing interests.

## Authors' contributions

JL conceived of and wrote the entire article.
