# “Correction: Practical aspects in the management of hypokalemic periodic paralysis”

**DOI:** 10.1186/1479-5876-12-198

**Published:** 2014-07-17

**Authors:** Jacob O Levitt

**Affiliations:** 1 President and Medical Director, Periodic Paralysis Association, 155 West 68th Street, #1732, New York, NY 10023, USA; 2 Department of Dermatology, The Mount Sinai Medical Center, New York, NY, USA

## Text

After the publication of this work [1], we noticed an error in Table 1. The dosage of potassium citrate monohydrate should be 1622 mg, not 540 mg as originally stated. The corrected table is included below.

**Table 1:** **(corrected)**: **Potassium salts and their milligram to milliequivalent (or millimole) relationships**

**Spoken Name**	**Chemical Formula**	**mg**	**mEq K**â€‰+â€‰**(or mM K**+**)**
Elemental potassium ion	K+	39.1	1
Potassium chloride (powder or tablets)	KCl	1500	20
Potassium bicarbonate (powder, tablets, or self-dissolving tablets)	KHCO_3_	650	6.5
Potassium citrate monohydrate	K_3_C_6_H_5_O_7_*H_2_O	1622	15 (i.e., 5 mEq of K_3_C_6_H_5_O_7_*H_2_O)
Potassium chloride 10% oral solution, 15 mL	KCl 10% oral solution	1500	20
Potassium chloride 20% oral solution, 15 mL	KCl 20% oral solution	3000	40
Potassium gluconate elixer, 15 mL	KC_6_H_11_O_7_	4680	20
Potassium gluconate tablets	KC_6_H_11_O_7_	500	2

The author apologises for this oversight.
